# Supplementary material for: How does the digital economy affect the green development of China’s industry?
Source: PLoS One. 2024 Sep 26;19(9):e0309590. doi: 10.1371/journal.pone.0309590 (PMC11426523; doi:10.1371/journal.pone.0309590)
Supplement: S1 Table — (PDF) [file pone.0309590.s001.pdf]

Supporting information

S1 Table. Results of bootstrapping mediation regression analysis

Mediator	Effect	Observed Coef.	Bootstrap Std. Err.	P>|z|	[95% conf. interval]
GTEC	Indirect effect	0.316	0.105	0.003	[0.1112, 0.5224]
	Direct effect	0.337	0.174	0.052	[-0.0035, 0.6767]
