# Supplementary material for: How does the digital economy affect the green development of China’s industry?
Source: PLoS One. 2024 Sep 26;19(9):e0309590. doi: 10.1371/journal.pone.0309590 (PMC11426523; doi:10.1371/journal.pone.0309590)
Supplement: Attachment — Submitted filename: Review report - PONE-D-24-11461(6.27).docx [file pone.0309590.s003.docx]

Review report - PONE-D-24-11461

How does the digital economy affect the green development of China's industry?

Major Points

1. The introduction emphasizes the shortcomings of existing research in exploring the impact of the digital economy on industrial green development, and points out that the existing literature lacks systematic research on how the digital economy promotes industrial green development, especially research on its internal mechanisms and spatial spillover effects.

2. Although the importance of the digital economy is mentioned in the introduction, there is a lack of detailed elaboration on how the digital economy specifically affects green development, and the logical relationship between the two is not fully demonstrated.

3. At the end of the introduction, the core issues and innovations of this study are clearly stated, and the unique contribution and practical significance of this study in the existing literature are highlighted.
4. The current summary section of the review lacks a systematic discussion of the shortcomings of existing research. It is recommended to further analyze where existing research is deficient and how these deficiencies affect the conduct of this study.
5. The argumentation chain is incomplete. There is a lack of sufficient argumentation and explanation for the citation of some research conclusions. For example, when mentioning "the important role of digital economy in reducing carbon emissions", you can add some specific mechanism explanations, such as resource optimization allocation, industrial structure upgrading, etc.
6. Inadequate independence of the assumed parts:

Each hypothesis should be formulated more independently and explicitly. The current hypothesis is partially mixed in the literature review, making it easy for readers to confuse the hypothesis with the content of the literature review. It is recommended that hypotheses be listed independently and provide concise theoretical support before each hypothesis.

7. The article uses Moran's I index to test the spatial correlation between the digital economy and industrial greening. It is recommended to provide more specific calculation formulas and steps, as well as an in-depth explanation of the results. Specifically, the construction method of the weight matrix can be shown, and the interpretation of Moran's I value and its application in the spatial Durbin model (SDM) can be analyzed.
8. Although Moran's I test and SDM model selection were conducted, there was no in-depth discussion on the substantive impact mechanism of spatial correlation and its specific application in the model. For example, how does the digital economy affect the green development of neighboring cities through spatial spillover effects? Why is the SDM model more suitable for exploring this spatial effect? It is recommended to consider these to increase your credibility.

9.文中提到了增加控制变量后模型拟合度（R2值）的增加，表明控制变量的选择是合理的。然而，具体控制变量的选择依据和它们在模型中的作用机制没有详细阐述。例如，控制变量如何与数字经济、工业绿色发展之间的关系联系起来？请给出具体解释。

9. The article mentions that the model fitting degree (R2 value) increases after adding control variables, indicating that the selection of control variables is reasonable. However, the basis for selecting specific control variables and their mechanism of action in the model are not elaborated in detail. For example, how do control variables relate to the relationship between the digital economy and industrial green development? Please give a specific explanation.
10. Although the intermediary effect of the digital economy in promoting industrial green development through green technology innovation is mentioned, there is a lack of detailed explanation of the definition, measurement method and specific mechanism of GTEC (green technology innovation) in the model. How to ensure that GTEC is a valid mediating variable? Were sensitivity analyzes conducted to verify the robustness of the mediating effects?
