# Supplementary material for: How does the digital economy affect the green development of China’s industry?
Source: PLoS One. 2024 Sep 26;19(9):e0309590. doi: 10.1371/journal.pone.0309590 (PMC11426523; doi:10.1371/journal.pone.0309590)
Supplement: Attachment — Submitted filename: Response to Reviewers.docx [file pone.0309590.s004.docx]

Responses to Reviewer Comments

**Ref: PONE-D-24-11461**

**Manuscript Title:**

**How does the digital economy affect the green development of China’s industry?**

We greatly appreciate the editor and the anonymous reviewers for their insightful comments and constructive suggestions, which immensely helped us improve the manuscript.

We have revised the paper accordingly. In this document, we respond to questions raised by the reviewers and explain what changes we have made. We hope the review panel finds the revised version satisfactory. All the changes can be seen in the revised manuscript and formatted in red.

In the following, we respond in detail to the comments from the review panel in a point-by-point manner. The key points raised by the reviewers are underlined; our responses and discussions are formatted in blue and bordered.

**Content**

[Response to Reviewer 1	2](#_Toc171709738)

[Response to Reviewer 2	4](#_Toc171709739)

# Response to Reviewer 1

**Comment 1**:

The introduction is not well designed. I suggest that this paper gives why this issue is being studied, how it is being studied, the results of the study, etc., it is necessary to state the main contributions, and how it differs from the rest of the literature.

Thank you so much for your careful check. The introduction is revised, the second paragraph describes why the problem is being studied, the third paragraph describes the research questions, and the fourth paragraph describes the three contributions of the paper.

**Comment 2**:

The abstract does not well written and should rewritten it according to this journal.

Thank you for your comment. Abstracts have been rewritten according to journal requirements.

**Comment 3**:

The authors have given some contributions, but these three contributions have been studied in the previous literature. Especially, I suggest authors conduct a compare with the existing papers.

Thank you for pointing out this problem in the manuscript. In order to provide a more in-depth comparison with existing papers, the literature review has been rewritten to include additional references to the literature, in addition to further refining and summarizing existing references, as described in the references section [7] [27] [48] [53] [54] [60] [61] [76]. The third and fourth paragraphs of the literature review section compare the existing papers.

**Comment 4**:

The theoretical analysis is confusing and lacks logic. The authors do not conduct an in-depth analysis of its influence mechanism around the topic of this paper.

Thank you for your comment. The content of the analysis of impact mechanisms has been rationalized and modified, as described in sections 3.1 and 3.2.

**Comment 5**:

I suggest that the authors should not simply list the literature, but summarize and refine it. In addition, it is recommended to cite some of the latest literature. These papers maybe useful for your revision: Does digital inclusive finance affect urban carbon emission intensity: Evidence from 285 cities in China. Cities. Seeing green: how does digital infrastructure affect carbon emission intensity? Energy Economics.

Thank you for your comment. Firstly, the literature review was completely revised to further compare and analyze the existing literature. Secondly, thank you for providing literature references that helped to refine the analysis of existing studies in this paper, so we have cited both of them, as described in references [27] and [48].

**Comment 6**:

The empirical analysis does not provide an in-depth discussion of its economic implications, that is, the results are not discussed from an economic perspective, including a comparative analysis with the existing literature.

Thank you for your comment. An analysis of the economic significance of the regression results has been added in section 5.3.3.

**Comment 7**:

Authors need to check the full text.

Thank you so much for your careful check. We carefully checked the full text and made changes to the following: the abstract section, paragraphs 2-4 of the introduction section, the literature review section, sections 3.1 and 3.2 of the research hypotheses section, section 4.2.3 on the measurement of other variables, and section 5.3.3 on the economic implications of the regression results.

# Response to Reviewer 2

The introduction emphasizes the shortcomings of existing research in exploring the impact of the digital economy on industrial green development, and points out that the existing literature lacks systematic research on how the digital economy promotes industrial green development, especially research on its internal mechanisms and spatial spillover effects.

Thank you for the affirmation.

**Comment 2:**

Although the importance of the digital economy is mentioned in the introduction, there is a lack of detailed elaboration on how the digital economy specifically affects green development, and the logical relationship between the two is not fully demonstrated.

Thank you for your comment. A discussion of the impact of the digital economy on green development has been added to the second paragraph of the introduction, which has been partially abridged and reordered for logical clarity.

**Comment 3**:

At the end of the introduction, the core issues and innovations of this study are clearly stated, and the unique contribution and practical significance of this study in the existing literature are highlighted.

Thank you for the affirmation.

**Comment 4:**

The current summary section of the review lacks a systematic discussion of the shortcomings of existing research. It is recommended to further analyze where existing research is deficient and how these deficiencies affect the conduct of this study.

Thank you for your advice. Firstly, the first and second paragraphs of the literature review section provide further refinement and comparative analysis. Then a discussion of existing research gaps and the implications of these gaps for this study is added in the third and fourth paragraphs.

**Comment 5**:

The argumentation chain is incomplete. There is a lack of sufficient argumentation and explanation for the citation of some research conclusions. For example, when mentioning "the important role of digital economy in reducing carbon emissions", you can add some specific mechanism explanations, such as resource optimization allocation, industrial structure upgrading, etc.

Thank you for your question. The findings of the cited studies are supplemented with arguments, as described in the first and second paragraphs of the literature review. For example, in the 10th line of the second paragraph of the literature review section, it is mentioned that "the digital economy plays an important role in reducing carbon emissions", followed by the 11th and 12th lines, in which the specific mechanism of the argument is developed, "the digital economy not only reduces carbon emissions directly, but also reduces carbon emissions through the optimisation of resource allocation, upgrading of industrial structure, reduction of energy intensity and transformation of energy structure [23-26,75]". intensity and energy structure transformation, etc. to reduce carbon emissions [23-26,75]". and further specified in lines 13-15.

**Comment 6**:

Inadequate independence of the assumed parts:

Each hypothesis should be formulated more independently and explicitly. The current hypothesis is partially mixed in the literature review, making it easy for readers to confuse the hypothesis with the content of the literature review. It is recommended that hypotheses be listed independently and provide concise theoretical support before each hypothesis.

Thank you for your question. Firstly, the mixed content existing between the research hypotheses and the literature review was deleted and modified; then the three hypotheses were listed independently, and the content of the theoretical support before the hypotheses was refined and perfected, for example, Hypothesis 1: The digital economy can promote the green development of industry. Before this hypothesis was put forward, since the digital economy was mostly measured from two aspects, namely, Internet development and digital financial development, theoretical support was provided from these two perspectives; on the one hand, Internet development is conducive to reducing energy consumption as well as reducing carbon emissions, thus facilitating the green development of industry. On the other hand, digital financial development is conducive to easing the financing constraints of enterprises and promoting their green innovation, thus favouring industrial green development. See sections 3.1, 3.2 and 3.3 for details.

**Comment 7**:

The article uses Moran's I index to test the spatial correlation between the digital economy and industrial greening. It is recommended to provide more specific calculation formulas and steps, as well as an in-depth explanation of the results. Specifically, the construction method of the weight matrix can be shown, and the interpretation of Moran's I value and its application in the spatial Durbin model (SDM) can be analyzed.

Firstly, the specific formulae and steps are described in Equation (6) and related content in 4.1.3.

Second, the construction of the weight matrix is given in equation (7) and related content.

Third, Moran's I values are analysed in the first paragraph of section 5.3.1. Fourthly, the application of Moran's I in the spatial Durbin model is shown in part 4.1.3. Since spatial econometric models can be used only when there is spatial correlation, Moran's I is used to examine whether there is spatial dependence between the digital economy and the industrial green total factor productivity before using the spatial econometric model, and the Moran's I takes a value ranging from -1 to 1. The closer to 0 the less spatial correlation, and the opposite the greater.

**Comment 8**:

Although Moran's I test and SDM model selection were conducted, there was no in-depth discussion on the substantive impact mechanism of spatial correlation and its specific application in the model. For example, how does the digital economy affect the green development of neighboring cities through spatial spillover effects? Why is the SDM model more suitable for exploring this spatial effect? It is recommended to consider these to increase your credibility.

Thank you for your question. Firstly, the content of part 5.3.3 has been modified to explain how the digital economy affects the green development of neighbouring cities through spatial spillover effects from both market and government perspectives.

Secondly, part 5.3.2 explains why the SDM model was chosen.

**Comment 9**:

The article mentions that the model fitting degree (R2 value) increases after adding control variables, indicating that the selection of control variables is reasonable. However, the basis for selecting specific control variables and their mechanism of action in the model are not elaborated in detail. For example, how do control variables relate to the relationship between the digital economy and industrial green development? Please give a specific explanation.

Thank you for your question. Firstly, the basis for the selection of the control variables is added with a specific explanation in the second paragraph of part 4.2.3.

Secondly, the mechanism by which the control variables work in the model is specified in the second paragraph of part 5.1.

**Comment 10**:

Although the intermediary effect of the digital economy in promoting industrial green development through green technology innovation is mentioned, there is a lack of detailed explanation of the definition, measurement method and specific mechanism of GTEC (green technology innovation) in the model. How to ensure that GTEC is a valid mediating variable? Were sensitivity analyzes conducted to verify the robustness of the mediating effects?

Thank you for your question. First, the definition of GTEC is added in lines 2-4 of the first paragraph of part 4.2.3, the measurement of GTEC is added in lines 5-6 of the first paragraph of part 4.2.3, and the detailed explanation of the specific mechanism is added and refined in part 3.2.

Second, to ensure that GTEC is a valid mediating variable, the paper employs a bootstrap test, the results of which are presented in Appendix Table S1, and the details of which are presented in the second paragraph of Part 5.2.
